# Molecular Characterization of Ahp2, a Lytic Bacteriophage of *Aeromonas hydrophila*

**DOI:** 10.3390/v13030477

**Published:** 2021-03-14

**Authors:** Jian-Bin Wang, Mei-Shiuan Yu, Tsai-Tien Tseng, Ling-Chun Lin

**Affiliations:** 1 Laboratory of Microbial Genetics, Institute of Medical Sciences, Tzu Chi University, No. 701, Sec. 3, Zhongyang Rd., Hualien 97004, Taiwan; kiyaky@gmail.com; 2 Department of Microbiology, School of Medicine, Tzu Chi University, No. 701, Sec. 3, Zhongyang Rd., Hualien 97004, Taiwan; meishiuan@gms.tcu.edu.tw; 3 Master Program in Microbiology and Immunology, School of Medicine, Tzu Chi University, No. 701, Sec. 3, Zhongyang Rd., Hualien 97004, Taiwan; 4 Department of Molecular and Cellular Biology, Kennesaw State University, 1000 Chastain Road, Kennesaw, GA 30144, USA; ttseng@kennesaw.edu

**Keywords:** *Aeromonas hydrophila*, *Aeromonas* phage, Ahp2, myophage

## Abstract

*Aeromonas hydrophila* is an opportunistic pathogen that infects fish, amphibians, mammals, and humans. This study isolated a myophage, vB_AhyM_Ahp2 (Ahp2), that lytically infects *A. hydrophila*. We observed that 96% of the Ahp2 particles adsorbed to *A. hydrophila* within 18 min. Ahp2 also showed a latent period of 15 min with a burst size of 142 PFU/cell. This phage has a linear double-stranded DNA genome of 47,331 bp with a GC content of 57%. At least 20 Ahp2 proteins were detected by SDS-polyacrylamide gel electrophoresis; among them, a 40-kDa protein was predicted as the major capsid protein. Sequence analysis showed that Ahp2 has a genome organization closely related to a group of *Aeromonas* phages (13AhydR10RR, 14AhydR10RR, 85AhydR10RR, phage 3, 32 Asp37, 59.1), which infect *Aeromonas hydrophila* and *Aeromonas salmonicida*. The tail module encompassing ORF27-29 in the Ahp2 genome was present in all *Aeromonas* phages analyzed in this study and likely determines the host range of the virus. This study found that Ahp2 completely lyses *A. hydrophila* AH300206 in 3.5 h at a MOI of 0.0001 and does not lysogenize its host. Altogether, these findings show that Ahp2 is a lytic *Aeromonas* phage and could be a candidate for therapeutic phage cocktails.

## 1. Introduction

*Aeromonas hydrophila* is a species of Gram-negative bacteria present in different environments as well as fish, invertebrates, birds, and humans [1]. This pathogen causes red-leg disease in frogs, hemorrhagic septicemia in fish, septic arthritis in calves, and gastroenteritis, septicemia, peritonitis, pneumonia, skin and soft tissue infections in humans [1,2,3]. Although antibiotics are commonly used to treat *A. hydrophila* infections, multidrug-resistant isolates have evolved in recent years [4,5,6] including those resistant to extended-spectrum beta-lactams and carbapenems [4,7,8,9,10], making the treatment of *A. hydrophila* infections challenging.

Bacteriophages have emerged as an attractive alternative for the control of bacterial pathogens resistant to antimicrobials [11]. In fact, phage therapy is expected to reduce the incidence of antimicrobial resistance [12,13] and has been successfully used in the context of many bacterial pathogens causing disease in animals and humans [14,15,16,17,18]. Although bacteriophages infecting *A. hydrophila* have been isolated including ahI, ah2, and ah3, the podophages Ahp1, MJG, and 25AhydR2PP [19,20,21], the shiphophages AhSzq-1, AhSzw-1, Akh-2 [22,23], the filamentous phage PM3 [24] and the myophages pAh1-C, pAh6-C, 13AhydR10PP, 14 AhydR10PP, 85 AhydR10PP, 50 AhydR10PP, 60 AhydR10PP, AH1, Aeh1, Aeh2, PM2, VTCCBPA6, CC2, and AhyVDH1 [15,21,25,26,27,28,29], their biological properties are only partially characterized. In a previous study, we identified a podophage of *A. hydrophila*, Ahp1, of the φKMV family [19]. Here, to expand the repertoire of phages available for targeting *A. hydrophila* therapeutic purposes, we isolated a new myophage from sewage and designated it as vB_AhyM_Ahp2 (Ahp2). In this study, we report the morphology, physiological characteristics, and results of its genome analysis.

## 2. Materials and Methods

### 2.1. Bacterial Strains and Growth Conditions 

The bacterial strains used in this study are listed in Table 1. *Staphylococcus aureus* NCTC8325 was cultured in tryptic soy broth (TSB) or TSB agar (Bacto, Mt. Pritchard, NSW, Australia) at 37 °C; the other bacteria were cultured in Luria Bertani (LB) broth and LB agar (Bacto) at 37 °C, except for *A. hydrophila* and *Xanthomonas campestris* pv. *campestris*, which were cultured at 30 °C and 28 °C, respectively. Bacterial growth was monitored via turbidimetry at 600 nm (OD_600_). For *A. hydrophila* AH300206, one OD unit corresponds to 1.2 × 10^8^ CFU/mL. The clinical and environmental isolates of *A. hydrophila* were classified by 16S rDNA sequencing using primers described elsewhere [30]. 

### 2.2. Phage Isolation and Host-Range Testing

Ahp2 was isolated from sewage. Phage isolation, plaque assays, and spot tests were conducted according to the methods reported previously [33]. For host-range testing, 5 μL phage lysate (1.0 × 10^7^ PFU/mL) was spotted on double-layered plates containing 100 μL different *A. hydrophila* strains (1.0 × 10^8^ CFU/mL). Clear zones were observed for susceptible hosts after an overnight incubation.

### 2.3. Isolation of Phage-Resistant Mutants and Determination of the Lysogenic Activity of Ahp2

The isolation of phage-resistant mutants and the determination of the lysogenic activity of Ahp2 were conducted according to the methods described earlier [34]. *A. hydrophila* AH300206 (106 CFU/mL) was mixed with 10^10^ PFU/mL Ahp2 in LB broth, and the mixture was incubated at room temperature for 10 min to allow phage adsorption. The mixture (400 µL) was added to 3 mL molten soft agar (0.7%) and poured onto LB plates. After incubation for two days at 30 °C, colonies grown on agar plates were regarded as phage-resistant mutants. After that, ten single colonies were separately picked and cultured in 5 mL LB broth at 30 °C overnight. The bacteria were again plated to isolate single colonies, which were then cultured in LB broth. This process was repeated five times to remove infective phage particles, and finally infected by Ahp2 to confirm its resistance. Bacterial DNA was then extracted for PCR analysis using the primers listed in Table 2. PCR was conducted at 94 °C for 2 min, 30 cycles of denaturation (94 °C, 30 s), annealing (64 °C, 5 s) and extension (72 °C, 30 s), and a final extension step at 72 °C for 5 min. PCR products were analyzed by agarose gel electrophoresis, followed by ethidium bromide staining and visualization under a UV trans-illuminator.

### 2.4. Phage Adsorption, One-Step Growth, and Purification 

Phage adsorption and one-step growth were examined according to the methods described previously [19]. Phage was purified using a protocol described elsewhere [33]. Phage lysates (200 mL; 1.0 × 10^10^ PFU/mL) were centrifuged at 7800× *g* for 10 min. The supernatants were passed through 0.45-μm membrane filters and centrifuged at 46,500× *g* (Beckman Coulter Avanti centrifuge, J25I rotor; Beckman Coulter, Brea, CA, USA) for 2 h at 4 °C. The pellets were suspended in 1.0 mL SM buffer (0.05 M Tris-HCl, pH 7.5, containing 0.1 M NaCl, 0.008 M MgSO_4_·7H_2_O, and 0.01% gelatin) and loaded onto a discontinuous CsCl gradient (ρ = 1.55, 1.50, 1.48, 1.45, and 1.40 g/cm^3^), followed by ultracentrifugation at 154,000× *g* for 3 h at 4 °C with an SW41Ti rotor in an Optima LE-80K Ultracentrifuge (Beckman Coulter). The phage particles were then recovered from the gradient, desalted with Amicon Ultra Centrifugal Filters (10,000 MWCO, Millipore, Burlington, MA, USA), and stored at 4 °C until use.

### 2.5. Phage DNA Isolation and Pulsed-Field Gel Electrophoresis 

The procedures described by Chang et al. [35] were used for isolating phage DNA and restriction enzyme digestion. Pulsed-field gel electrophoresis (PFGE) was performed as described previously [36], using the CHEF-DR III System (Bio-Rad Laboratories, Hercules, CA, USA) at 9 °C in 0.5 × Tris-borate-EDTA buffer, pH 8.0 at 6 V/cm with pulse ramps from 3.5 to 4 s for 19.5 h. The Midrange IPFG Marker (New England Biolabs, Ipswich, MA, USA) was used as the molecular size standard.

### 2.6. Protein Analyses

Phage particles purified by CsCl-gradient centrifugation were mixed with a gel electrophoresis sample buffer (100 mM Tris-HCl, pH 6.8, 4% SDS, 0.2% bromophenol blue, 20% glycerol, 200 mM dithiothreitol), boiled for 10 min, and subjected to SDS-polyacrylamide gel electrophoresis (8–16% gradient). Protein bands were visualized using InstantBlue (Expedeon Protein Solutions Ltd., Cambridge, UK). To determine the identity of the protein in a gel, protein bands were excised and subjected to peptide mass fingerprinting using a Microflex instrument (Bruker Corporation, Billerica, MA, USA) for de novo peptide analysis by matrix-assisted laser desorption ionization-time of flight (MALDI-TOF) mass spectrometry. The spectra were recorded in the linear mode in a detection range of 500–3500 m/z and subsequently analyzed using the flexAnalysis software version 3.4 (Bruker Corporation). The data were searched using the MASCOT peptide mass fingerprinting search program, against a local database of possible peptide spectra deduced from the Ahp2 genome sequence. 

### 2.7. Electron Microscopy

To observe Ahp2, a 10 μL phage suspension (1.0 × 10^11^ PFU/mL) was applied onto the surface of a formvar-coated grid (400 mesh copper grids), negatively stained with 2% uranyl-acetate for 30 s, and then examined under a Hitachi H-7500 transmission electron microscope (Tokyo, Japan) operated at 80 kV. Fifteen phage images were examined for measuring the size of the phage particles.

### 2.8. Whole-Genome Sequencing and In Silico Analysis

Ahp2 DNA was sequenced with a Next Generation Sequencing system (Solexa technology, Illumina, San Diego, CA, USA) using the paired-end method. Potential open reading frames (ORFs) were predicted using the GeneMarkS software and the MPI Bioinformatics Toolkit [37,38]. Translated ORFs were compared to the non-redundant GenBank protein database, using BLASTP (http://blast.ncbi.nlm.nih.gov/Blast.cgi (02/07/2019), and HHPred [39]. The presence of transmembrane domains was verified with the TMHMM software [40]. Whole genome alignments among all *Aeromonas* phages were conducted by EasyFig 2.2.3 [41]. 

### 2.9. Nucleotide Sequence Accession Number

The genome sequence of Ahp2 was deposited in GenBank under the accession number KX455876.

## 3. Results

### 3.1. Morphology and Biological Properties of Ahp2

**Figure 1:**
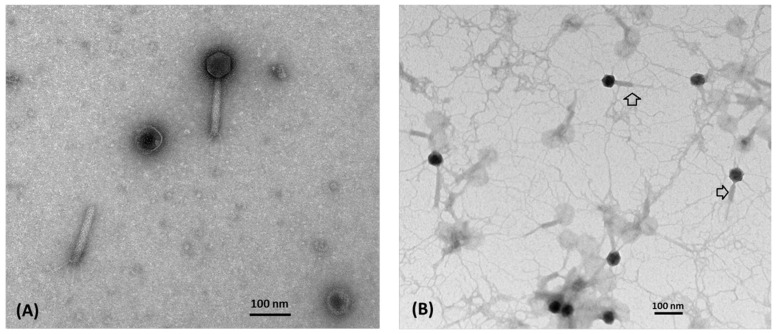
Morphology of Ahp2. Ahp2 was negatively stained with 2% uranyl acetate and examined under a transmission electron microscope. A total of 15 phage images were examined. Representative images show the phage particles with uncontracted (**A**) and contracted (**B**) tails (Arrow) Bar: 100 nm.

Water samples collected from sewage, wastewater treatment plants, and aquariums were screened for the presence of lytic phages via spot tests using *A. hydrophila* ATCC7966 and three clinical isolates (AH19288, AH60114, and AH300206). One phage that lysed *A. hydrophila* AH300206 was isolated and designated as vB_AhyM_Ahp2 (Ahp2). Electron microscopy revealed a typical myophage morphology. The phage has an isometric head (69.4 ± 1.6 nm in diameter), a long contractile tail of 132.4 ± 5.0 × 14.7 ± 2.0 nm in length, a baseplate (25.5 ± 2.3 nm in width), and tail fibers (Figure 1). 

Different conditions were tested in the context of phage propagation. We infected a culture of *A. hydrophila* AH300206 (200 mL of LB broth in a 500 mL flask) at the exponential phase (0.6 OD600 unit) with Ahp2 using a MOI of 0.0001. After infection, the turbidity of the bacterial culture was measured every 30 min. The turbidity of the culture initially increased, but started to decrease gradually at hour 2; complete lysis of the bacteria was observed at hour 3.5. The infection ultimately resulted in 3.0 × 10^10^ PFU/mL of phage progeny. Since the MOI used was low, complete lysis suggests that Ahp2 is a lytic phage.

**Figure 2:**
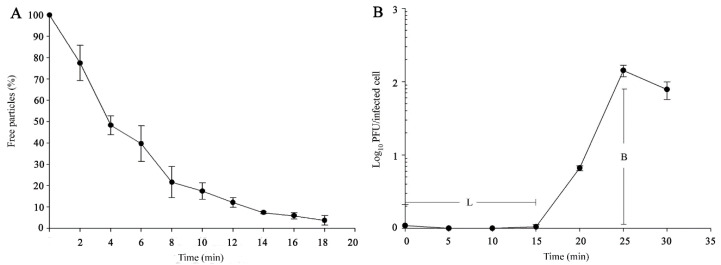
Biological properties of Ahp2. (**A**) Adsorption rate in the context of the *A. hydrophila* AH300206. Un-adsorbed phages in supernatants were assayed. (**B**) One-step growth of Ahp2 in the context of *A. hydrophila* AH300206. L: latent period, B: burst size. Values represent the mean of three independent experiments. Bar: standard deviation.

Importantly, approximately 96% of Ahp2 phages adsorbed to *A. hydrophila* AH300206 (Figure 2A) within 18 min. To study the growth profile of Ahp2, a one-step growth curve in the context of *A. hydrophila* AH300206 was determined. As shown in Figure 2B, Ahp2 exhibited a latent period of 15 min, and a replication period of 25 min. The average burst size was estimated to be 142 PFU/infected cell.

### 3.2. Ahp2 Infects a Wide Range of Environmental A. hydrophila Strains

A total of 42 *A. hydrophila* strains (Table 1) were used for spot tests with Ahp2. Results showed that 25 strains or 59.5% of the strains tested (AH300206, H2, H4-H7, H12-H21, H23-H25, H27, H28, H30-H32, and H34) manifested clearing zones, while the others were resistant to Ahp2 infection. Meanwhile, the phage did not infect *Acinetobacter baumannii* ATCC17978, *Escherichia coli* DH5, *Klebsiella pneumoniae* Kp6, *Staphylococcus aureus* NCTC8325, *Vibrio parahaemolyticus* VP93, *V. harveyi* BAA-1117, and *Xanthomonas campestris* pv. Campestris P20H (Table 1).

### 3.3. General Properties of the Ahp2 Genome

The genome length of Ahp2 has been estimated as 47 kb according to PFGE (Figure S1A). The sequencing results confirmed this estimation, revealing that the Ahp2 genome consists of 47,331 bp. The genome has a GC content of 57%, lower than that of sequenced *A. hydrophila* strains (ca. NC_008570.1, 61%, NZ_CP006579.1, 62%). Ahp2 genomic DNA had two BglII-cutting sites. Thus, upon digestion with BglII, three fragments would be generated if the genomic DNA is linear, while two fragments would be obtained if it was circular. In our experiments, three BglII fragments were visualized in the agarose gel (Figure S1B), suggesting that the Ahp2 genome is linear.

**Figure 3:**
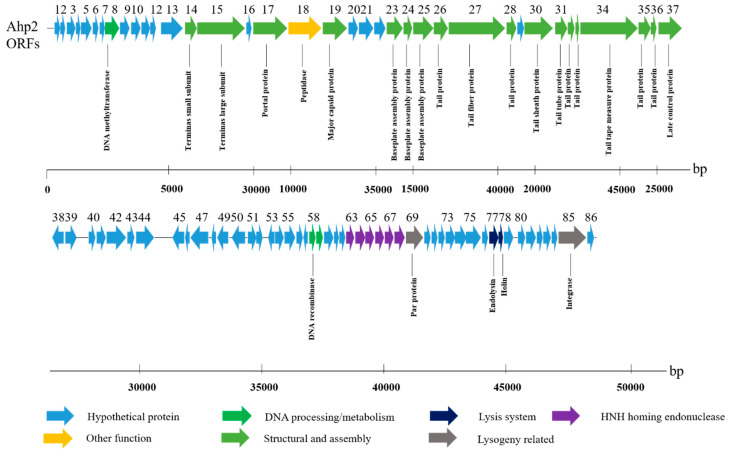
Annotated map of the Ahp2 genome. The predicted ORFs are numbered. Predicted gene functions are shown in different colors.

Moreover, the results revealed that the Ahp2 genome encodes 86 ORFs, occupying 91.8% of the entire genome. Based on sequence analysis, almost all ORFs showed best-matched orthologs closely related to proteins from other *Aeromonas* phages (Table S1). Sequence analysis showed that most ORFs were located on the plus-strand; only nine ORFs were transcribed from the opposite direction (Figure 3). All ORFs began with AUG, except for ORFs 25, 53, and 78, which used UUG; ORFs 38, 46, 61, 73, 79, 84, and 85 used GUG as initiation codons (Figure 3). The genome of Ahp2 is organized into several modules that contain genes encoding proteins involved in packaging, morphogenesis, replication/transcription, and host-cell lysis as well as structural proteins. The genes in the structural module (ORFs 14–37) encode a terminase, a portal protein, a coat protein, a baseplate assembly protein, tail fiber-proteins, a tail sheath protein, a tail tube protein, a tail tape measure protein, and a late control protein (Figure 3). Moreover, sequencing analysis revealed that Ahp2 did not seem to encode any proteins related to toxicity, pathogenicity, or antibiotic resistance.

### 3.4. Genome Comparison of Ahp2 and Similar Aeromonas Phages

**Figure 4:**
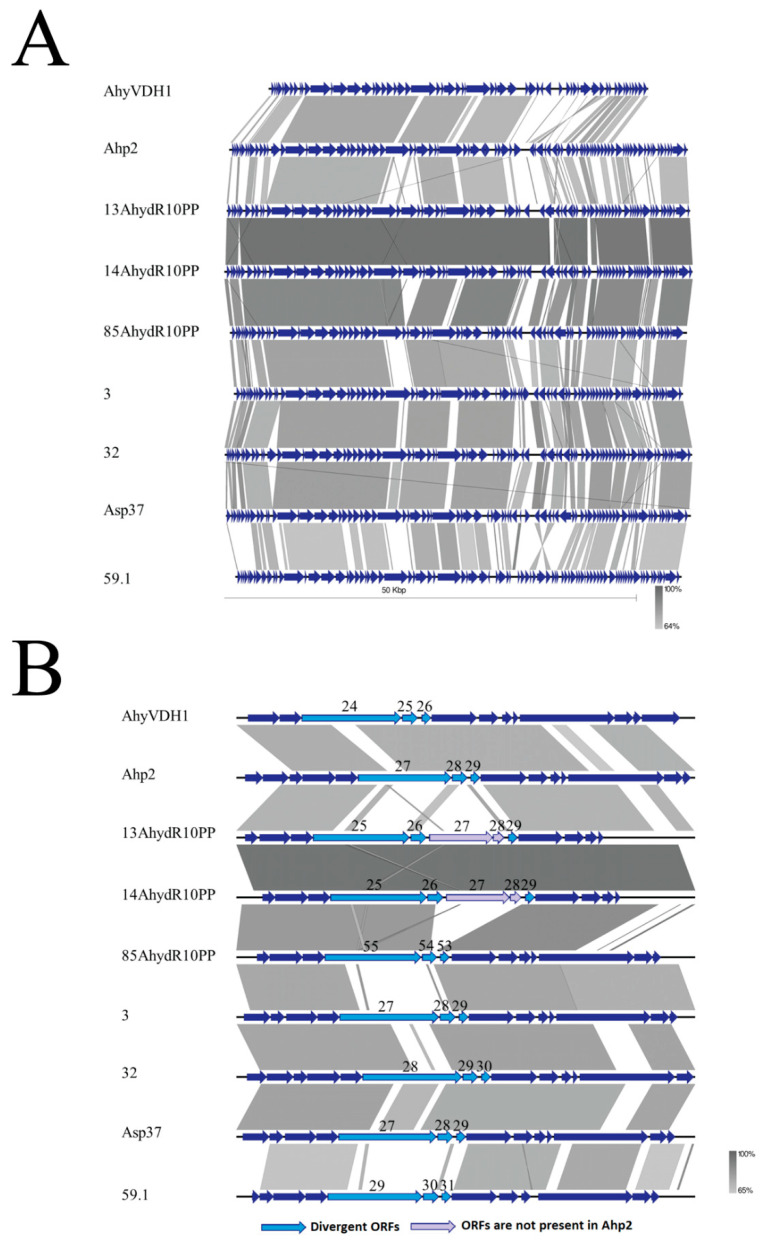
Comparison of the phage genomes. (**A**) The whole genome and (**B**) the region containing the ORF27-ORF29 orthologs are illustrated and compared. The genomes of Ahp2 and *A. hydrophila* phages (AhyVDH1, 13AhydR10PP, 14AhydR10PP, 85AhydR10PP) and *A. salmonicida* phages (3, 32, Asp37, 59.1) were aligned and compared using EasyFig. The identity cutoff was 64%. Number indicates the ORF number of the phages.

The genome sequence of Ahp2 analyzed by using Blast 2 with default parameters showed that three *A. salmonicida* phages (ASP37, phage 3, phage 32) [42] had 36%–38% query cover and 74%–75% identity to Ahp2. Another *A. salmonicida* phage, phage 59.1, with low sequence identity with the three phages described above, has a short sequence matched with Ahp2 (Figure 4A). A group of unclassified phages that infects *A. hydrophila* including 13AhydR10PP, 14AhydR10PP, 85AhydR10PP, and AhyVDH1 [21,29] was also compared with Ahp2. Phages 13AhydR10PP and 14AhydR10PP were similar to each other with 96% query cover and 96% identity. We found that these two phages only had 18% query cover and 78% identity with Ahp2, lower than the query cover and sequence identity of *A. salmonicida* phages (ASP37, phage 3, phage 32). Phage 85AhydR10PP, having 78% query cover and 89% identity with 13AhydR10PP or 14AhydR10PP, has 39% query cover and 75% identity with Ahp2. Phage AhyVDH1 showed 77% identity from 46% coverage of Ahp2 genomic sequence, but lacks an 8156 bp fragment corresponding to the right-end of Ahp2 (Figure 4A).

To gain insights into the relationship between the tail module and host range, genomic position 13,000 to 25,000, encompassing ORFs 23–34 in Ahp2 and the same modules in other *Aeromonas* phages, were zoomed in for further analysis. The orthologs of ORF27, ORF28, and ORF29 in Ahp2, which encode two tail fiber proteins and a protein of unknown functions, respectively, were present in all the *Aeromonas* phages that we examined in this study. The protein sequences of the orthologs were compared by protein Blast 2, and the results showed that the ORFs described above in these regions were relatively divergent with 42% to 77% identity (Table S2), which may contribute to the host range differences among the phages. We also found that two additional ORFs were present in this region in 13AhydR10PP and 14AhydR10PP, which were absent in Ahp2 and the others (Figure 4B).

### 3.5. Gene Products and Their Functions

#### 3.5.1. Morphogenesis and Structural Genes

The ORFs 14 and 15 of Ahp2 encode a small subunit (terS) and a large subunit (terL) of a phage terminase, containing a DUF1441 superfamily domain (aa 11-152, pfam07278) and a Terminase_GpA superfamily domain (aa 42-655, pfam05876), respectively. The proteins encoded by these genes are likely responsible for the processing and packaging of the replicated phage genome concatemers into the mature virion, as is in the known cases [43]. ORF17, with a Phage_portal_2 domain (aa 19-361, pfam05136), encodes a protein similar to the portal protein in the phages of the lambda family, responsible for the formation of a ring, enabling the passage of the DNA passage, packaging, and ejection, and for the formation of the junction between the coat and tail proteins [44,45]. Additionally, ORF19 likely encodes the major capsid protein, containing a Phage_cap_E domain (aa 17-331, pfam03864) (see Section 3.6).

ORFs 23-25 of Ahp2 likely encode tail proteins including a potential phage baseplate assembly protein, with Phage_base_V (aa 10-117, pfam04717), W (aa 1-106, PHA02516), and J (aa 4-276, PHA02568) domains. ORF26 contains a Tail_P2_I domain (aa 22-158, pfam09684). ORF27 encodes a protein with a DUF3751 domain (aa 14-102, pfam12571) and has a 31% similarity to the phage hinge connector in the long tail fiber distal connector protein of the *Aeromonas* phage CC2 (accession number: YP_007010398.1). ORF28 encodes a protein showing 43% similarity to the phage tail protein of *Aeromonas enteropelogenes* (accession number: WP_042069835.1). ORF30 encodes a putative phage tail sheath protein with an FI domain (aa 24-396, PHA02560). ORF31 likely encodes the phage tail tube protein containing a Phage_tube domain (aa 5-165, pfam04985). These two proteins are similar to the components of the contractile tail, a hallmark of the phages of the Myoviridae family [46]. In fact, our electron microscopy study verified the presence of such a contractile tail in Ahp2 (Figure 1). ORF32 of Ahp2 encodes a protein with a Phage_TAC_7 domain (aa 4-86, pfam10109), which is annotated as a phage tail assembly protein. ORF33 encodes a protein containing a Phage_P2_GpE domain (aa 5-40, pfam06528), annotated as a phage tail protein. ORF34 encodes a protein containing a tape_meas_TP901 domain (aa 199-545, TIGR01760), showing 43% similarity to the phage tail tape measure protein of *Aeromonas aquatica* (accession number: WP_034514527.1). ORF35 encodes a putative phage tail formation protein with a Phage_P2_GpU domain (aa 29-160, pfam06995). ORF36 encodes a putative phage tail protein with a Phage_tail_X domain (aa 8-62, pfam05489). Finally, ORF37 encodes a protein with a Phage_GPD domain (aa 104-310, annotated as a putative phage late control protein.

#### 3.5.2. Genes Involved in Replication and Recombination

Ahp2 ORF58 encodes a protein containing an SR_ResInv domain (aa 1-20, cd03768) and an HTH_Hin_like domain (aa 32-73, cd00569) typically present in members of the serine recombinase (SR) family, suggesting that the ORF58 protein is involved in DNA recombination. ORF59 encodes a protein showing 51% similarity to a hypothetical protein of Marinobacter sp. ELB17 (accession number: WP_007352549.1) and 39% similarity to the hypothetical helicase of the prophage CP-933R of *Salmonella enterica* B182 (accession number: AFH45012.1), which is likely involved in the initiation of DNA replication. Importantly, the region upstream of ORF69 in Ahp2, which contains ORFs 63-68, is unique among *Aeromonas* phages (13AhydR10PP, 14AhydR10PP, 85AhydR10PP, 3, 32, Asp37, 59.1) (Figure 3 and Figure 4A); these six ORFs were annotated as putative HNH homing endonucleases according to their predicted motifs (Table S1).

#### 3.5.3. Lysis and Lysogeny Genes

During the release of their progeny, enzymes related to the lytic mechanism are used to lyse the bacterial host; members of the classical holin-endolysin lysis system have been found in many phages [45]. Endolysins can be divided into five main classes [46]; one of them comprises the endopeptidases, also known as endolysins or peptidoglycan hydrolases which cleave the peptide moiety. ORF77 of Ahp2 encodes a protein containing a Peptidase_M15_3 domain (aa 17-111, pfam08291) with a sequence similar to that of an endopeptidase. Moreover, located downstream of the predicted endopeptidase-coding gene, ORF77, is ORF78, which likely encodes a small type-II holin, with two transmembrane domains that are typically present in many holins [47]. Ahp2 ORF85 encodes a putative phage integrase with a domain similar to the INT_P4_C domain (aa 220-384, cd00801) of the integrase in P4-like lysogenic phages; of note, the ORF85 protein is 26% and 22% similar to the integrase of the lambda-like temperate phages HK022 (NC_002166.1) and phi80 (NC_021190.1), respectively.

#### 3.5.4. Other Open Reading Frames (ORFs)

In addition to the ORFs described above, the protein encoded by ORF08 is a putative DNA adenine methyltransferase (DNA MTase) with high degrees of identity with the homologs from *Photobacterium aquimaris* (60%; accession number: WP_065189998.1) and many *Aeromonas* species such as *A. jandaei* (60%; accession number: WP_042031719.1). Additionally, the protein encoded by ORF09, containing a PRK11675 domain (aa 16-51), shares 49% similarity with a conserved hypothetical protein of *A. jandaei* phage (accession number: WP_042031722.1). ORF18 also encodes a protein with an S49_Sppa_36K_type domain (aa 64-276, cd07022), which is a putative serine peptidase. 

Last but not least, the protein product of ORF69 has two motifs similar to those of a ParA-like protein [48], which often collaborates with ParB to ensure the partition of the correct copy number of the bacterial chromosome and plasmids into daughter cells. One of these two motifs is located between aa 26-33 (QXXXXGKS), which is similar to the phosphate-binding loop (GXXXXGK-T/S) [49], and the other, between aa 102 and 106 (LIIVD), is similar to the Walker A and B motifs.

### 3.6. Characterization of the Most Abundant Protein in the Ahp2 Virion

**Figure 5:**
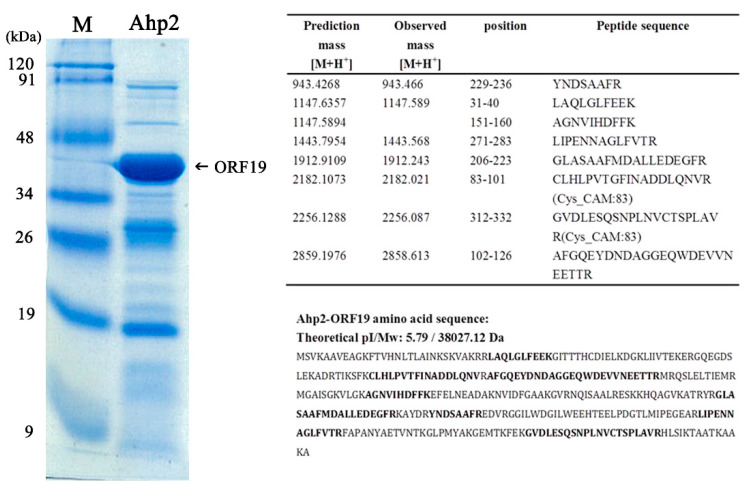
Ahp2 virion proteins. Phage particles (5 × 1011 PFU) were boiled in sample buffer (20 μL) and subjected to SDS-polyacrylamide gel electrophoresis (8–16% gradient). The arrow indicates ORF19, which has been identified by peptide mass fingerprinting as the major coat protein of Ahp2. The peptide fingerprints matched the deduced sequence of ORF19 shown in the right panel. Cysteine residues in the protein were converted to carbamidomethyl-cysteine (Cys_CAM) with iodoacetamide. Lane M, pre-stained middle range protein marker. Boldfaced sequence: peptide identified by MALDI-TOF.

SDS-polyacrylamide gel electrophoresis revealed that Ahp2 virions contain at least 20 proteins (Figure 5). A band with an apparent molecular mass of 40 kDa was most abundant, which is likely to be the major capsid protein of Ahp2. MALDI-TOF analysis of this protein revealed seven observed peptides with observed mass [M+H+] close to the predicted mass of the amino acid sequence deduced from Trysin-digested ORF19, length range from eight to 25 amino acids. The possible peptides matched were listed in the right panel in Figure 5. The results verified that the 40-kDa is encoded by ORF19.

### 3.7. Detection of Lysogen in Ahp2

**Figure 6:**
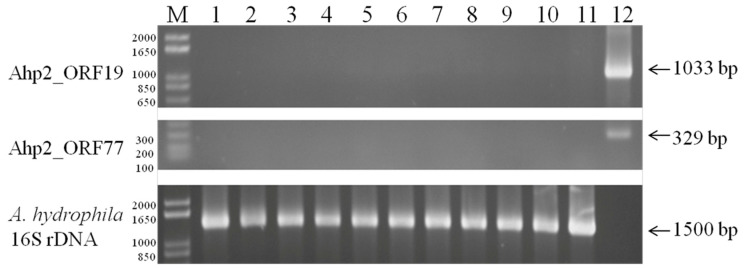
PCR detection of Ahp2 ORF19 and ORF77 in phage-resistant mutants of *A. hydrophila* AH300206. DNA templates were prepared from 10 Ahp2-resistant mutants of *A. hydrophila* AH300206 (lanes 1 to 10), *A. hydrophila* AH300206 (lane 11), and Ahp2 (lane 12) for the amplification of ORF19, PRF77, and 16S rRNA. “M”: molecular size marker (1 kb Plus DNA ladder, Invitrogen).

As sequence analysis revealed that the Ahp2 contains an integrase gene, this prompted us to examine whether Ahp2 was a lysogenic phage. As is generally known that a lysogen is immune to phage infection, *A. hydrophila* AH300206 mutants that were resistant to phage infection were isolated according to the method described in the Materials and Methods [33]. After the DNA was extracted from the mutants, the presence of Ahp2_ORF19 (coat protein) and Ahp2_ORF77 (endopeptidase) in the mutants were detected by PCR. We analyzed the DNA from 10 phage-resistance colonies by PCR, but were unable to detect the presence of Ahp2 DNA (Figure 6), suggesting that despite the presence of an integrase gene, the phage does not lysogenize *A. hydrophila* AH300206.

## 4. Discussion

In this study, a myophage of *A. hydrophila* designated as vB_AhyM_Ahp2 (Ahp2) was isolated and characterized. Approximately 96% of Ahp2 phages adsorbed to *A. hydrophila* AH300206 within 18 min (Figure 2A), which was much slower than the observed rate of 2 min for phage Ahp1 [19]. The latent period was about 15 min, and the average burst size was estimated to be 142 PFU/infected cell (Figure 2B), slightly higher than that for Ahp1 (112 PFU/infected cell) and much higher than those for Aeh1 (17 PFU/infected cell), Aeh2 (92 PFU/infected cell), pAh1-C (60 PFU/infected cell), and pAh6-C (10 PFU/infected cell) [15,25], but lower than a closely related phage AhyVDH1 (274PFU/infected cell) [29]. Of note, the latent period of Ahp2 was shorter than the other *A. hydrophila* myophages including Aeh1 (39 min), Aeh2 (52 min), pAh1-C (30 min), pAh6-C (20 min), and AhyVDH1 (50 min) [15,25,29].

Among the *A. hydrophila* phages, AhyVDH1 has a narrow host range, infecting only one strain [29]. Kazimierczak et al. [21] found that 13AhydR10PP, 14AhydR10PP, and 85AhydR10PP infected at least two *Aeromonas* species including *A. hydrophila*, *Aeromonas sobria* or *Aeromonas salmonicida*, and 51%, 51%, and 43% of the 49 strains tested, respectively, showing that these three phages have a broad host range. Phages 3, 32, and Asp37 have an intermediate host range, infecting 10–30 strains of *A. salmonicida* including subspecies *Aeromonas smithia*, *Aeromonas masoucida*, and *Aeromonas pectinolytica* (65 tested strains) [21,42]; another phage that infects *A. salmonicida*, 59.1, had a narrow host range, infecting only five strains. This study found that Ahp2 infected 25 out 42 (60%) strains, most of which were environmental isolates, except *A. hydrophila* AH300206, which was isolated from a hospital. Previously, we found that Ahp1 infected only six strains that are listed in Table 1 (ATCC 7966, H6, H10, H23, H30, and H32) [19], showing that Ahp2 has a host range broader than Ahp1. Among these six strains, H6, H23, H30, and H32 can be infected by both Ahp1 and Ahp2. As *Aeromonas* phages have a different host range, a cocktail consisting of different types of phages will be necessary to facilitate phage therapy. 

Tail fiber and tail related proteins are known to be associated with host specificity [50,51]. Our comparative genome analysis among tail modules manifested the most divergent ORFs (corresponding to ORF27-29 of Ahp2). Genomic analysis revealed that the ORF27-ORF29 region in Ahp2 was also present in other *Aeromonas* phages, although their identity ranged from 42 to 77% (Table S2). As ORF27, ORF28, and ORF29 encode proteins in the tail structures, the sequence diversity may contribute to the host range difference. The two additional ORFs in the same region in 13AhydR10PP and 14AhydR10PP may also be important to the infection of their hosts. 

The overall genomic arrangement of Ahp2 is closely related to that of a group of *Aeromonas* phages infecting *A. hydrophila* as above-mentioned (Figure 4A) [21,42], except that the region at the right-end of Ahp2 genome including ORF55-86 is uniquely absent in AhyVDH1 (Figure 4A) [29]. This region contains 32 ORFs including mostly hypothetical proteins and two putative lysis-related ORFs (ORF77-78). The lack of this region, especially a lysis gene, does not seem to influence viral release by AhyVDH1 [29]. The predicted lysis function in Ahp2 and the other seven *Aeromonas* phages need to be elucidated by further genetic studies.

Previous studies have predicted that *A. salmonicida* (3, 32, Asp37, 59.1) and *A. hydrophila* (13AhydR10PP, 14AhydR10PP, 85AhydR10PP) phages are lysogenic, based on the presence of an integrase gene in their genome [21,42]. In the current study, we demonstrated that Ahp2 is probably not a lysogenic phage (Figure 6). Although Ahp2 contains an integrase gene, the other genes necessary for lysogenization are absent. The presence of an incomplete set of lysogenic genes were also observed in many lytic phages including *Pseudomonas plecoglossicida* phage PPpW-3, *A. salmonicida* phage 56, and the *Iodobacter* sp. Phage φPLPE [34,42,52]. 

Phage therapy has gained attention recently due to the development of resistance of pathogens to antibiotics [53,54]. In fact, phage preparations have already been used clinically and in agriculture for treating bacterial infections [55,56,57]. Furthermore, many tests have been conducted experimentally to evaluate the efficacy of phage therapy on *Aeromonas* infections that showed promising results [58,59,60]. As bacterial resistance to phage infection is a major issue for phage therapy, isolating new phage strains is crucial. We combined experimental and bioinformatic approach to characterize a newly isolated phage, Ahp2. Our study demonstrates that Ahp2 and similar *Aeromonas* phages have divergent tail protein ORFs, which may explain why these phages usually do not infect a broad range of *Aeromonas* [21,29,42]. The isolation and characterization of Ahp2 will facilitate the development of a useful preparation for treating *Aeromonas* infections.

## 5. Conclusions

In this study, a lytic bacteriophage of *A. hydrophila*, Ahp2, was isolated from sewage. Our results suggest that Ahp2 is a myophage, exclusively lytic, which could be useful for the treatment of *A. hydrophila* infections.

## Figures and Tables

**Table 1:** Bacterial strains used in this study.

Strain	Descriptions	Reference or Source
*A. hydrophila*		
ATCC7966	Type strain, Ap ^r^	ATCC
ATCC43414	Type strain, Ap ^r^	ATCC
AH19288	Clinical isolate from Buddhist Tzu Chi General Hospital, Ap ^r^	[19]
AH60114, AH300206	Clinical isolates from Hualien Armed Forces General Hospital, Ap ^r^	[19]
Hua-1, Hua-2	Fish isolates from Hualien Animal and Plant Disease Control Center, Ap ^r^	[19]
H1 to H35	Environmental isolates, Ap ^r^	[19]
*Acinetobacter baumannii*		
ATCC17978	Type strain, Ap ^r^	ATCC
*Escherichia coli*		
DH5α	F^− φ^80d*lac*ZΔM15Δ(*lacZYA*-*argF*) U169 *recA1 endA1 hsdR*17 (r_k_^−^, m_k_^+^) *pho*A *supE44* λ^−^ *thi*-*1 gyrA96 relA1*	[31]
*Klebsiella pneumonia*		
Kp-6	Clinical isolate, Ap ^r^	N. T. Lin *^a^*
*Staphylococcus aureus*		
NCTC8325	Type strain, Ap ^r^	NCTC (National Collection of Type Cultures)
*Vibrio parahaemolyticus*		
VP93	Clinical isolate, Ap ^r^	M. S. Yu *^a^*
*Vibrio harveyi*		
BAA-1117	*lux*N::tn5Kan	ATCC
*Xanthomonas campestris* pv. *campestris*		
P20H	Nonmucoid mutant, Ap ^r^	[32]

*^a^* Tzu Chi University, Hualien, Taiwan; ^r^: resistance.

**Table 2:** PCR primers used in this study.

Genes	Primer Name	Primer Sequence	Product Size (bp)	Reference
16S rDNA	27F	5′-AGAGTTTGATCMTGGCTCAG	1500	[30]
	1492R	5′-TACGGYTACCTTGTTACGACTT		
Ahp2_ORF19 (coat protein)	MCP_F	5′-GCGTAAAAGCTGCCGTAGAA	1033	This study
	MCP_R	5′-GCTTTAGCTGCCTTGGTTGC		
Ahp2_ORF77 (endopeptidase)	77_F	5′-AAGACGTAAAGCTGCGCTGC	329	This study
	77_R	5′-TTAGTAGTCAAAAACGATGC		

## Data Availability

The data presented in this study are available in this article and supplementary material here.

## Supplementary Materials

The following are available online at https://www.mdpi.com/1999-4915/13/3/477/s1, Figure S1. Estimation of genome size and restriction analysis of Ahp2 by pulsed-field gel electrophoresis. Table S1. Genome annotation of *Aeromonas hydrophila* phage Ahp2. Table S2. Proteins encoded by the genes in the ORF23-ORF37 region in Ahp2 and their orthologs encoded by other *Aeromonas* phages.
