# Supplementary material for: Molecular Characterization of Ahp2, a Lytic Bacteriophage of Aeromonas hydrophila
Source: Viruses. 2021 Mar 14;13(3):477. doi: 10.3390/v13030477 (PMC8001559; doi:10.3390/v13030477)
Supplement: Supplementary file 1 [file viruses-13-00477-s001.zip › Table S1.docx]

**Table S1**. **Genome annotation of *Aeromonas hydrophila* phage Ahp2**

| *orf* | Start | Stop | G+C (%) | Length (aa) | Mol Wt (kDa) | % Identity to best homologs | Best homologs | Motif accession | Protein function/motif |
| --- | --- | --- | --- | --- | --- | --- | --- | --- | --- |
| 01 | 328 (ATG) | 555 (TAA) | 56.6 | 75 | 8.4 | 27/48 (56%) | APU00810.1  (Aeromonas phage 59.1) |  | Hypothetical protein |
| 02 | 555 (ATG) | 737 (TAA) | 55.7 | 60 | 6.4 | 23/58 (40%) | APU01143.1  (Aeromonas phage 32) |  | Hypothetical protein |
| 03 | 750 (ATG) | 1094 (TGA) | 53.9 | 114 | 13.5 | 67/114 (59%) | APU00728.1  (Aeromonas phage Asp37) |  | Hypothetical protein |
| 04 | 1091 (ATG) | 1258 (TGA) | 53.6 | 55 | 6.2 | 31/54 (57%) | APU01145.1  (Aeromonas phage 32) |  | Hypothetical protein) |
| 05 | 1251 (ATG) | 1694 (TGA) | 57.7 | 147 | 16.7 | － |  |  | No similarity |
| 06 | 1720 (ATG) | 1944 (TAG) | 59.1 | 74 | 8.2 | 31/71 (44%) | APU01148.1  (Aeromonas phage 32) |  | Hypothetical protein |
| 07 | 1946 (ATG) | 2155 (TGA) | 54.8 | 69 | 7.5 | － |  |  | No similarity |
| 08 | 2152 (ATG) | 2733 (TGA) | 58.1 | 193 | 22.0 | 149/193 (77%) | APU01149.1  (Aeromonas phage 32) | pfam02384 | putative DNA adenine methyltransferase/ N6_Mtase domain |
| 09 | 2730 (ATG) | 3110 (TAG) | 58.8 | 126 | 14.1 | 90/122 (74%) | APU00399.1  (Aeromonas phage 3) | cl08198 | Conserved hypothetical protein/PRK11675 domain |
| 10 | 3178 (ATG) | 3579 (TGA) | 59.2 | 133 | 14.7 | 45/118 (38%) | WP_033129404.1  (Aeromonas aquatica) |  | Hypothetical protein |
| 11 | 3579 (ATG) | 3929 (TGA) | 57.6 | 116 | 12.4 | 50/83 (60%) | APU00821.1  (Aeromonas phage 59.1) |  | Hypothetical protein |
| 12 | 3922 (ATG) | 4128 (TGA) | 56.0 | 68 | 7.1 | 41/68 (60%) | APU01153.1  (Aeromonas phage 32) |  | Hypothetical protein |
| 13 | 4347 (ATG) | 5282 (TGA) | 57.3 | 311 | 33.8 | 155/312 (50%) | APU01154.1  (Aeromonas phage 32) | pfam10544 | Hypothetical protein/  T5orf172 |
| 14 | 5352 (ATG) | 5837 (TAA) | 56.0 | 161 | 18.1 | 114/161 (71%) | APU01155.1  (Aeromonas phage 32) | cl06347 | Terminase small subunit/  DUF1441 superfamily |
| 15 | 5837 (ATG) | 7909 (TAA) | 58.4 | 690 | 78.0 | 539/693 (78%) | APU01156.1  (Aeromonas phage 32) | cl21617 | Terminase large subunit/  Terminase_GpA superfamily |
| 16 | 7952 (ATG) | 8155 (TGA) | 57.8 | 67 | 7.5 | 50/67 (75%) | APU01157.1  (Aeromonas phage 32) |  | head-tail adaptor Ad1 |
| 17 | 8203 (ATG) | 9669 (TGA) | 58.0 | 488 | 54.7 | 364/489 (74%) | APU01158.1  (Aeromonas phage 32) | pfam05136 | Portal protein/  Phage_portal_2 |
| 18 | 9675 (ATG) | 11099 (TAA) | 58.7 | 474 | 49.4 | 376/473 (79%) | APU01159.1  (Aeromonas phage 32) | cd07022 | head maturation protease/  S49_Sppa_36K_type |
| 19 | 11153 (ATG) | 12193 (TAA) | 55.8 | 346 | 38.2 | 299/344 (87%) | APU00408.1  (Aeromonas phage 3) | pfam03864 | major head protein/  Phage_cap_E |
| 20 | 12193 (ATG) | 12570 (TAA) | 55.6 | 125 | 13.9 | 89/126 (71%) | APU01161.1  (Aeromonas phage 32) |  | Hypothetical protein |
| 21 | 12557 (ATG) | 13162 (TAA) | 55.5 | 201 | 22.5 | 127/199 (64%) | APU00746.1  (Aeromonas phage Asp37) |  | neck protein Ne1 |
| 22 | 13228 (ATG) | 13698 (TGA) | 59.9 | 156 | 17.3 | 118/156 (76%) | APU00411.1  (Aeromonas phage 3) |  | Hypothetical protein |
| 23 | 13695 (ATG) | 14375 (TAA) | 59.8 | 226 | 22.8 | 164/234 (70%) | APU01164.1  (Aeromonas phage 32) | pfam04717 | Baseplate assembly protein V/  Phage_base_V superfamily |
| 24 | 14400 (ATG) | 14744 (TGA) | 53.6 | 114 | 12.5 | 92/114 (81%) | APU00413.1  (Aeromonas phage 3) | PHA02516 | Baseplate assembly protein W/  GPW_gp25 superfamily |
| 25 | 14731 (TTG) | 15603 (TGA) | 59.5 | 290 | 31.9 | 218/289 (75%) | APU01166.1  (Aeromonas phage 32) | PHA02568 | Baseplate assembly protein J/  Baseplate_J superfamily |
| 26 | 15600 (ATG) | 16178 (TGA) | 57.2 | 192 | 21.6 | 160/190 (84%) | APU01167.1  (Aeromonas phage 32) | pfam09684 | Tail protein/  Tail_P2_I |
| 27 | 16175 (ATG) | 18628 (TAA) | 56.6 | 817 | 87.4 | 441/869 (51%) | APU00752.1  (Aeromonas phage Asp37) | pfam12571 | Tail fiber protein/  DUF3751 superfamily |
| 28 | 18641 (ATG) | 19051 (TAA) | 59.1 | 136 | 15.4 | 105/136 (77%) | APU01169.1  (Aeromonas phage 32) |  | Tail fibers |
| 29 | 19125 (ATG) | 19382 (TAA) | 58.2 | 85 | 9.2 | 25/49 (51%) | APU00754.1  (Aeromonas phage Asp37) |  | Hypothetical protein |
| 30 | 19392 (ATG) | 20600 (TAA) | 58.2 | 402 | 43.8 | 380/402 (95%) | APU01171.1  (Aeromonas phage 32) | PHA02560 | Tail sheath/  FI domain |
| 31 | 20645 (ATG) | 21154 (TAA) | 56.3 | 169 | 18.7 | 148/169 (88%) | APU01172.1  (Aeromonas phage 32) | pfam04985 | Major tail tube protein/  Phage_tube |
| 32 | 21220 (ATG) | 21489 (TAA) | 58.9 | 89 | 10.1 | 77/89 (87%) | APU01173.1  (Aeromonas phage 32) | pfam10109 | Tail protein/Phage_TAC_7 |
| 33 | 21510 (ATG) | 21644 (TAA) | 49.6 | 44 | 5.2 | 36/44 (82%) | APU00422.1  (Aeromonas phage 3) | pfam06528 | Tail protein/ Phage_P2_GpE superfamily |
| 34 | 21683 (ATG) | 24187 (TAA) | 59.0 | 834 | 88.7 | 555/817 (68%) | APU01175.1  (Aeromonas phage 32) | TIGR01760 | Tail length tape-measure protein/ tape_meas_TP901 |
| 35 | 24187 (ATG) | 24681 (TGA) | 58.8 | 164 | 17.8 | 129/163 (79%) | APU00424.1  (Aeromonas phage 3) | pfam06995 | Tail protein/  Phage_P2_GpU |
| 36 | 24678 (ATG) | 24893 (TAA) | 51.4 | 71 | 8.3 | 55/71 (77%) | APU00761.1  (Aeromonas phage Asp37) | pfam05489 | Tail protein/  Phage_tail_X |
| 37 | 24903 (ATG) | 25904 (TGA) | 58.9 | 333 | 36.4 | 235/333 (71%) | APU01178.1  (Aeromonas phage 32) | cl15796 | late control protein GpD/  Phage_GPD superfamily |
| 38 | 25945 (GTG) | 26412 (TAG) | 59.2 | 155 | 16.5 | 16/46 (35%) | GAN00870.1 |  | Sn-1,2-diacylglycerol cholinephosphotransferas-e (Mucor ambiguus) |
| 39 | 26458 (ATG) | 26931 (TAA) | 59.9 | 157 | 17.4 | 77/152 (51%) | APU00427.1  (Aeromonas phage 3) |  | Hypothetical protein |
| 40 | 27439 (ATG) | 27708 (TAG) | 53.0 | 89 | 9.9 | － |  |  | No similarity |
| 41 | 27742 (ATG) | 28110 (TGA) | 56.4 | 122 | 13.9 | 59/88 (67%) | APU00429.1  (Aeromonas phage 3) |  | Hypothetical protein |
| 42 | 28097 (ATG) | 28915 (TGA) | 58.2 | 272 | 30.3 | 108/278 (39%) | APU00850.1  (Aeromonas phage 59.1) |  | Hypothetical protein  (unique in Aeromonas phages) |
| 43 | 28960 (ATG) | 29247 (TGA) | 53.5 | 95 | 11.4 | 43/98 (44%) | APU00851.1  (Aeromonas 59.1) |  | Hypothetical protein |
| 44 | 29410 (ATG) | 30180 (TAG) | 58.0 | 256 | 27.9 | 70/182 (38%) | APU01185.1  (Aeromonas phage 32) |  | Hypothetical protein |
| 45 | 30969 (ATG) | 31460 (TAG) | 56.3 | 163 | 18.8 | 67/156 (43%) | APU00774.1  (Aeromonas phage Asp37) |  | Hypothetical protein |
| 46 | 31469 (GTG) | 31633 (TGA) | 53.3 | 54 | 6.2 | 14/43 (33%) | WP_053583340.1 |  | Hypothetical protein |
| 47 | 31630 (ATG) | 32403 (TGA) | 59.6 | 257 | 28.0 | 96/236 (41%) | APU00439.1  (Aeromonas phage 3) |  | Hypothetical protein |
| 48 | 32531 (ATG) | 32692 (TAA) | 51.9 | 53 | 6.0 | 31/53 (58%) | APU01191.1  (Aeromonas phage 32) |  | Hypothetical protein |
| 49 | 32706 (ATG) | 33170 (TGA) | 55.7 | 154 | 17.0 | 64/155 (41%) | APU00855.1  (Aeromonas phage 59.1) |  | Hypothetical protein |
| 50 | 33278 (ATG) | 33844 (TGA) | 58.7 | 188 | 21.3 | 94/177  (53%) | APU00779.1  (Aeromonas phage Asp37) |  | Hypothetical protein |
| 51 | 33940 (ATG) | 34284 (TGA) | 55.7 | 114 | 13.3 | 47/105  (45%) | APU01194.1  (Aeromonas phage 32) |  | Hypothetical protein |
| 52 | 34277 (ATG) | 34516 (TAA) | 56.7 | 79 | 9.1 | 63/79 (80%) | APU01196.1  (Aeromonas phage 32) |  | Hypothetical protein |
| 53 | 34746 (TTG) | 35009 (TAA) | 46.6 | 87 | 9.9 | － |  |  | No similarity |
| 54 | 35012 (ATG) | 35368 (TAA) | 58.8 | 118 | 13.2 | 67/111 (60%) | APU00864.1  (Aeromonas phage 59.1) |  | Hypothetical protein |
| 55 | 35397 (ATG) | 35837 (TAA) | 54.4 | 146 | 17.1 | － |  |  | No similarity |
| 56 | 35850 (ATG) | 36095 (TGA) | 52.9 | 81 | 9.2 | 40/77 (52%) | APU00865.1  (Aeromonas phage 59.1) |  | Hypothetical protein |
| 57 | 36182 (ATG) | 36364 (TGA) | 62.8 | 60 | 6.2 | 19/46 (41%) | AOY74097.1  (Arthrobacter sp. ZXY-2) |  | Hypothetical protein |
| 58 | 36410 (ATG) | 36640 (TAA) | 58.9 | 76 | 8.3 | 66/75 (88%) | APU01199.1  (Aeromonas phage 32) | cd03768 | putative DNA invertase/ SR_ResInv/  HTH_Hin_like |
| 59 | 36640 (ATG) | 36900 (TAA) | 53.3 | 86 | 9.3 | 44/86 (51%) | WP_007352549.1  (Marinobacter sp. ELB17 ) |  | Hypothetical helicase |
| 60 | 37906 (ATG) | 37298 (TGA) | 58.0 | 130 | 14.6 | 83/127 (65%) | APU00785.1  (Aeromonas phage Asp37) |  | Hypothetical protein |
| 61 | 37295 (GTG) | 37477 (TGA) | 55.7 | 60 | 6.9 | 23/57 (40%) | WP_054021819.1  (Ideonella sakaiensis) |  | Hypothetical protein |
| 62 | 37474 (ATG) | 37707 (TGA) | 54.7 | 77 | 8.9 | 56/76 (74%) | APU01201.1  (Aeromonas phage 32) |  | Hypothetical protein |
| 63 | 37704 (ATG) | 38018(TGA) | 59.1 | 104 | 11.2 | 45/102 (44%) | APU00787.1  (Aeromonas phage Asp37) | smart00497 | putative HNH homing endonuclease/  IENR1 |
| 64 | 38015 (ATG) | 38419 (TGA) | 56.3 | 134 | 14.6 | 73/124 (59%) | APU00872.1  (Aeromonas phage 59.1) | cl21459 | putative HNH homing endonuclease/HTH superfamily |
| 65 | 38389 (ATG) | 39748 (TAA) | 59.4 | 119 | 13.4 | 71/117 (61%) | APU00873.1  (Aeromonas phage 59.1) | cl21459 | putative HNH homing endonuclease/HTH superfamily |
| 66 | 38753 (ATG) | 39106 (TGA) | 57.9 | 117 | 12.8 | 45/115 (39%) | APU00874.1  (Aeromonas phage 59.1) |  | putative HNH homing endonuclease |
| 67 | 39103 (ATG) | 39450 (TGA) | 60.9 | 115 | 12.1 | 48/115 (42%) | APU00875.1  (Aeromonas phage 59.1) |  | putative HNH homing endonuclease |
| 68 | 39444 (ATG) | 39860 (TGA) | 57.0 | 137 | 15.3 | 75/127 (59%) | APU00876.1  (Aeromonas phage 59.1) | cl21459 | putative HNH homing endonuclease |
| 69 | 39948 (ATG) | 40670 (TAA) | 58.2 | 240 | 26.2 | 140/216 (65%) | APU00457.1  (Aeromonas phage 3) | cd02042 | ParA-like partition protein/ParAB_family |
| 70 | 40673 (ATG) | 40921 (TGA) | 57.8 | 82 | 9.1 | 43/81 (53%) | APU00794.1  (Aeromonas phage Asp37) |  | Hypothetical protein |
| 71 | 40921 (ATG) | 41154 (TGA) | 51.7 | 77 | 8.4 | 42/77 (55%) | APU00460.1  (Aeromonas phage 3) |  | Hypothetical protein |
| 72 | 41151 (ATG) | 41414 (TAA) | 57.6 | 87 | 9.9 | 40/83 (48%) | APU00459.1  (Aeromonas phage 3) |  | Hypothetical protein |
| 73 | 41401 (GTG) | 41781 (TGA) | 55.4 | 126 | 14.6 | 32/84 (38%) | APU883.1  (Aeromonas phage 59.1) | cl11819 | Hypothetical protein/  LigD_N superfamily |
| 74 | 41769 (ATG) | 42260 (TGA) | 57.1 | 163 | 18.2 | 59/117 (50%) | APU1213.1  (Aeromonas phage 32) |  | Hypothetical protein |
| 75 | 42257(ATG) | 42871 (TGA) | 61.1 | 204 | 23.2 | 54/184 (29%) | APU01214.1  (Aeromonas phage 32) |  | Hypothetical protein |
| 76 | 42871 (ATG) | 43113 (TGA) | 57.2 | 80 | 9.2 | 58/80 (73%) | APU00800.1  (Aeromonas phage Asp37) |  | Hypothetical protein |
| 77 | 43194 (ATG) | 43559 (TAA) | 56.0 | 121 | 13.7 | 108/121 (89%) | APU01216.1  (Aeromonas phage 32) | pfam08291 | Endolysin/  Peptidase_M15_3 superfamily |
| 78 | 43549 (TTG) | 43731 (TGA) | 56.8 | 60 | 6.6 | － |  |  | putative holing |
| 79 | 43724 (GTG) | 44110 (TAG) | 58.7 | 128 | 14.3 | 18/49 (37%) | XP_010669359.1  (Beta vulgaris subsp. vulgaris) |  | Hypothetical protein |
| 80 | 44264 (ATG) | 44557 (TAA) | 53.4 | 97 | 10.5 | 47/77 (61%) | APU01217.1  (Aeromonas phage 32) |  | transcriptional regulator |
| 81 | 44550 (ATG) | 44957 (TAA) | 58.6 | 135 | 14.9 | 73/127 (57%) | APU00803.1  (Aeromonas phage Asp37) |  | Hypothetical protein |
| 82 | 44971 (ATG) | 45201 (TGA) | 54.1 | 76 | 8.9 | 44/64 (69%) | APU00804.1  (Aeromonas phage Asp37) |  | Hypothetical protein |
| 83 | 45290 (ATG) | 45589 (TGA) | 57.7 | 99 | 11.4 | 52/99 (53%) | APU01220.1  (Aeromonas phage 32) |  | Hypothetical protein |
| 84 | 45586 (GTG) | 45798 (TAA) | 59.2 | 70 | 7.7 | 46/70 (66%) | APU00470.1  (Aeromonas phage 3) |  | Hypothetical protein |
| 85 | 45801 (GTG) | 46976 (TGA) | 56.8 | 391 | 44.5 | 290/391 (74%) | APU00471.1  (Aeromonas phage 3) | cd00801 | Integrase/ INT_P4_C |
| 86 | 46973 (ATG) | 47254 (TGA) | 55.0 | 93 | 10.3 | 41/77 (53%) | APU00472.1  (Aeromonas phage 3) |  | Hypothetical protein |
