# Supplementary material for: Molecular Characterization of Ahp2, a Lytic Bacteriophage of Aeromonas hydrophila
Source: Viruses. 2021 Mar 14;13(3):477. doi: 10.3390/v13030477 (PMC8001559; doi:10.3390/v13030477)
Supplement: Supplementary file 1 [file viruses-13-00477-s001.zip › TableS2.docx]

**Table S2.** Proteins encoded by the genes in the ORF23-ORF37 region in Ahp2 and their orthologs encoded by other *Aeromonas* phages

| **ORF number** | **Ahp2 ORFs Accession** | **AhyVDH1 ORFs Accession** | **%identity** | **Annotation** |
| --- | --- | --- | --- | --- |
| ORF23 | ANZ52205.1_23 | QIZ02625.1_20 | 67.7 | Baseplate assembly protein V/  Phage_base_V superfamily |
| ORF24 | ANZ52206.1_24 | QIZ02626.1_21 | 80.7 | Baseplate assembly protein W/  GPW_gp25 superfamily |
| ORF25 | ANZ52207.1_25 | QIZ02627.1_22 | 84.8 | Baseplate assembly protein J/  Baseplate_J superfamily |
| ORF26 | ANZ52208.1_26 | QIZ02628.1_23 | 90.2 | Tail protein/Tail_P2_I |
| ORF27 | ANZ52209.1_27 | QIZ02629.1_24 | 54.8 | Tail fiber protein/  DUF3751 superfamily |
| ORF28 | ANZ52210.1_28 | QIZ02630.1_25 | 71.5 | Tail fibers |
| ORF29 | ANZ52211.1_29 | QIZ02631.1_26 | 56.5 | Hypothetical protein |
| ORF30 | ANZ52212.1_30 | QIZ02632.1_27 | 86.3 | Tail sheath/  FI domain |
| ORF31 | ANZ52213.1_31 | QIZ02633.1_28 | 93.5 | Major tail tube protein |
| ORF32 | ANZ52214.1_32 | QIZ02634.1_29 | 84.3 | Tail protein/Phage_TAC_7 |
| ORF33 | ANZ52215.1_33 | QIZ02635.1_30 | 79.5 | Tail protein/ Phage_P2_GpE superfamily |
| ORF34 | ANZ52216.1_34 | QIZ02636.1_31 | 55.3 | Tail length tape-measure protein/ tape_meas_TP901 |
| ORF35 | ANZ52217.1_35 | QIZ02637.1_32 | 77.6 | Tail protein |
| ORF36 | ANZ52218.1_36 | QIZ02638.1_33 | 76.1 | Tail protein/Phage_tail_X |
| ORF37 | ANZ52219.1_37 | QIZ02639.1_34 | 75.2 | late control protein GpD/  Phage_GPD superfamily |

| **ORF number** | **Ahp2 ORFs Accession** | **13AhydR10PP ORFs Accession** | **%identity** | **Annotation** |
| --- | --- | --- | --- | --- |
| ORF23 | ANZ52205.1_23 | AWH14523.1_21 | 71.681 | Baseplate assembly protein V/  Phage_base_V superfamily |
| ORF24 | ANZ52206.1_24 | AWH14524.1_22 | 84.956 | Baseplate assembly protein W/  GPW_gp25 superfamily |
| ORF25 | ANZ52207.1_25 | AWH14525.1_23 | 75.368 | Baseplate assembly protein J/  Baseplate_J superfamily |
| ORF26 | ANZ52208.1_26 | AWH14526.1_24 | 83.684 | Tail protein/Tail_P2_I |
| ORF27 | ANZ52209.1_27 | AWH14527.1_25 | 54.706 | Tail fiber protein/  DUF3751 superfamily |
| ORF28 | ANZ52210.1_28 | AWH14528.1_26 | 50.735 | Tail fibers |
|  |  | AWH14529.1_27 |  |  |
|  |  | AWH14530.1_28 |  |  |
| ORF29 | ANZ52211.1_29 | AWH14531.1_29 | 42.353 | Hypothetical protein |
| ORF30 | ANZ52212.1_30 | AWH14532.1_30 | 88.831 | Tail sheath/  FI domain |
| ORF31 | ANZ52213.1_31 | AWH14533.1_31 | 85.799 | Major tail tube protein |
| ORF32 | ANZ52214.1_32 | AWH14534.1_32 | 69.412 | Tail protein/Phage_TAC_7 |
| ORF33 | ANZ52215.1_33 | AWH14535.1_33 | 77.273 | Tail protein/ Phage_P2_GpE superfamily |
| ORF34 | ANZ52216.1_34 | AWH14536.1_34 | 55.755 | Tail length tape-measure protein/ tape_meas_TP901 |
| ORF35 | ANZ52217.1_35 | AWH14537.1_35 | 82.209 | Tail protein |
| ORF36 | ANZ52218.1_36 | AWH14538.1_36 | 73.239 | Tail protein/Phage_tail_X |
| ORF37 | ANZ52219.1_37 | AWH14539.1_37 | 68.769 | late control protein GpD/  Phage_GPD superfamily |

| **ORF number** | **Ahp2 ORFs Accession** | **14AhydR10PP ORFs Accession** | **%identity** | **Annotation** |
| --- | --- | --- | --- | --- |
| ORF23 | ANZ52205.1_23 | AWH15321.1_21 | 71.239 | Baseplate assembly protein V/  Phage_base_V superfamily |
| ORF24 | ANZ52206.1_24 | AWH15322.1_22 | 84.956 | Baseplate assembly protein W/  GPW_gp25 superfamily |
| ORF25 | ANZ52207.1_25 | AWH15323.1_23 | 75.61 | Baseplate assembly protein J/  Baseplate_J superfamily |
| ORF26 | ANZ52208.1_26 | AWH15324.1_24 | 83.684 | Tail protein/Tail_P2_I |
| ORF27 | ANZ52209.1_27 | AWH15325.1_25 | 54.706 | Tail fiber protein/  DUF3751 superfamily |
| ORF28 | ANZ52210.1_28 | AWH15326.1_26 | 48.175 | Tail fibers |
|  |  | AWH15327.1_27 |  |  |
|  |  | AWH15328.1_28 |  |  |
| ORF29 | ANZ52211.1_29 | AWH15329.1_29 | 42.353 | Hypothetical protein |
| ORF30 | ANZ52212.1_30 | AWH15330.1_30 | 89.61 | Tail sheath/  FI domain |
| ORF31 | ANZ52213.1_31 | AWH15331.1_31 | 85.799 | Major tail tube protein |
| ORF32 | ANZ52214.1_32 | AWH15332.1_32 | 67.059 | Tail protein/Phage_TAC_7 |
| ORF33 | ANZ52215.1_33 | AWH15333.1_33 | 77.273 | Tail protein/ Phage_P2_GpE superfamily |
| ORF34 | ANZ52216.1_34 | AWH15334.1_34 | 55.635 | Tail length tape-measure protein/ tape_meas_TP901 |
| ORF35 | ANZ52217.1_35 | AWH15335.1_35 | 82.209 | Tail protein |
| ORF36 | ANZ52218.1_36 | AWH15336.1_36 | 74.648 | Tail protein/Phage_tail_X |
| ORF37 | ANZ52219.1_37 | AWH15337.1_37 | 69.069 | late control protein GpD/  Phage_GPD superfamily |

| **ORF number** | **Ahp2 ORFs Accession** | **85AhydR10PP ORFs Accession** | **%identity** | **Annotation** |
| --- | --- | --- | --- | --- |
| ORF23 | ANZ52205.1_23 | AWH15051.1_59 | 70.796 | Baseplate assembly protein V/  Phage_base_V superfamily |
| ORF24 | ANZ52206.1_24 | AWH15050.1_58 | 86.607 | Baseplate assembly protein W/  GPW_gp25 superfamily |
| ORF25 | ANZ52207.1_25 | AWH15049.1_57 | 76.307 | Baseplate assembly protein J/  Baseplate_J superfamily |
| ORF26 | ANZ52208.1_26 | AWH15048.1_56 | 83.684 | Tail protein/Tail_P2_I |
| ORF27 | ANZ52209.1_27 | AWH15047.1_55 | 55.952 | Tail fiber protein/  DUF3751 superfamily |
| ORF28 | ANZ52210.1_28 | AWH15046.1_54 | 55.882 | Tail fibers |
| ORF29 | ANZ52211.1_29 | AWH15045.1_53 | 45.882 | Hypothetical protein |
| ORF30 | ANZ52212.1_30 | AWH15044.1_52 | 89.87 | Tail sheath/  FI domain |
| ORF31 | ANZ52213.1_31 | AWH15043.1_51 | 85.799 | Major tail tube protein |
| ORF32 | ANZ52214.1_32 | AWH15042.1_50 | 69.412 | Tail protein/Phage_TAC_7 |
| ORF33 | ANZ52215.1_33 | AWH15041.1_49 | 77.273 | Tail protein/ Phage_P2_GpE superfamily |
| ORF34 | ANZ52216.1_34 | AWH15040.1_48 | 66.307 | Tail length tape-measure protein/ tape_meas_TP901 |
| ORF35 | ANZ52217.1_35 | AWH15039.1_47 | 82.822 | Tail protein |
| ORF36 | ANZ52218.1_36 | AWH15038.1_46 | 73.239 | Tail protein/Phage_tail_X |
| ORF37 | ANZ52219.1_37 | AWH15037.1_45 | 68.769 | late control protein GpD/  Phage_GPD superfamily |

| **ORF number** | **Ahp2 ORFs Accession** | **Phage 3 ORFs Accession** | **%identity** | **Annotation** |
| --- | --- | --- | --- | --- |
| ORF23 | ANZ52205.1_23 | APU00412.1_23 | 66.239 | Baseplate assembly protein V/  Phage_base_V superfamily |
| ORF24 | ANZ52206.1_24 | APU00413.1_24 | 80.702 | Baseplate assembly protein W/  GPW_gp25 superfamily |
| ORF25 | ANZ52207.1_25 | APU00414.1_25 | 76.471 | Baseplate assembly protein J/  Baseplate_J superfamily |
| ORF26 | ANZ52208.1_26 | APU00415.1_26 | 84.375 | Tail protein/Tail_P2_I |
| ORF27 | ANZ52209.1_27 | APU00416.1_27 | 63.333 | Tail fiber protein/  DUF3751 superfamily |
| ORF28 | ANZ52210.1_28 | APU00417.1_28 | 61.481 | Tail fibers |
| ORF29 | ANZ52211.1_29 | APU00418.1_29 | 48.98 | Hypothetical protein |
| ORF30 | ANZ52212.1_30 | APU00419.1_30 | 89.552 | Tail sheath/  FI domain |
| ORF31 | ANZ52213.1_31 | APU00420.1_31 | 85.207 | Major tail tube protein |
| ORF32 | ANZ52214.1_32 | APU00421.1_32 | 78.161 | Tail protein/Phage_TAC_7 |
| ORF33 | ANZ52215.1_33 | APU00422.1_33 | 81.818 | Tail protein/ Phage_P2_GpE superfamily |
| ORF34 | ANZ52216.1_34 | APU00423.1_34 | 52.581 | Tail length tape-measure protein/ tape_meas_TP901 |
| ORF35 | ANZ52217.1_35 | APU00424.1_35 | 79.141 | Tail protein |
| ORF36 | ANZ52218.1_36 | APU00425.1_36 | 76.056 | Tail protein/Phage_tail_X |
| ORF37 | ANZ52219.1_37 | APU00426.1_37 | 69.67 | late control protein GpD/  Phage_GPD superfamily |

| **ORF number** | **Ahp2 ORFs Accession** | **Phage 32 ORFs Accession** | **%identity** | **Annotation** |
| --- | --- | --- | --- | --- |
| ORF23 | ANZ52205.1_23 | APU01164.1_24 | 70.085 | Baseplate assembly protein V/  Phage_base_V superfamily |
| ORF24 | ANZ52206.1_24 | APU01165.1_25 | 84.211 | Baseplate assembly protein W/  GPW_gp25 superfamily |
| ORF25 | ANZ52207.1_25 | APU01166.1_26 | 75.433 | Baseplate assembly protein J/  Baseplate_J superfamily |
| ORF26 | ANZ52208.1_26 | APU01167.1_27 | 84.211 | Tail protein/Tail_P2_I |
| ORF27 | ANZ52209.1_27 | APU01168.1_28 | 48.66 | Tail fiber protein/  DUF3751 superfamily |
| ORF28 | ANZ52210.1_28 | APU01169.1_29 | 77.206 | Tail fibers |
| ORF29 | ANZ52211.1_29 | APU01170.1_30 | 46.939 | Hypothetical protein |
| ORF30 | ANZ52212.1_30 | APU01171.1_31 | 94.527 | Tail sheath/  FI domain |
| ORF31 | ANZ52213.1_31 | APU01172.1_32 | 87.574 | Major tail tube protein |
| ORF32 | ANZ52214.1_32 | APU01173.1_33 | 86.517 | Tail protein/Phage_TAC_7 |
| ORF33 | ANZ52215.1_33 | APU01174.1_34 | 83.333 | Tail protein/ Phage_P2_GpE superfamily |
| ORF34 | ANZ52216.1_34 | APU01175.1_35 | 67.931 | Tail length tape-measure protein/ tape_meas_TP901 |
| ORF35 | ANZ52217.1_35 | APU01176.1_36 | 82.759 | Tail length tape-measure protein/ tape_meas_TP901 |
| ORF36 | ANZ52218.1_36 | APU01177.1_37 | 74.648 | Tail protein/Phage_tail_X |
| ORF37 | ANZ52219.1_37 | APU01178.1_38 | 70.571 | late control protein GpD/  Phage_GPD superfamily |

| **ORF number** | **Ahp2 ORFs Accession** | **Asp37 ORFs Accession** | **%identity** | **Annotation** |
| --- | --- | --- | --- | --- |
| ORF23 | ANZ52205.1_23 | APU00748.1_23 | 67.949 | Baseplate assembly protein V/  Phage_base_V superfamily |
| ORF24 | ANZ52206.1_24 | APU00749.1_24 | 76.316 | Baseplate assembly protein W/  GPW_gp25 superfamily |
| ORF25 | ANZ52207.1_25 | APU00750.1_25 | 76.103 | Baseplate assembly protein J/  Baseplate_J superfamily |
| ORF26 | ANZ52208.1_26 | APU00751.1_26 | 82.812 | Tail protein/Tail_P2_I |
| ORF27 | ANZ52209.1_27 | APU00752.1_27 | 50.748 | Tail fiber protein/  DUF3751 superfamily |
| ORF28 | ANZ52210.1_28 | APU00753.1_28 | 59.701 | Tail fibers |
| ORF29 | ANZ52211.1_29 | APU00754.1_29 | 51.02 | Hypothetical protein |
| ORF30 | ANZ52212.1_30 | APU00755.1_30 | 89.055 | Tail sheath/  FI domain |
| ORF31 | ANZ52213.1_31 | APU00756.1_31 | 85.207 | Major tail tube protein |
| ORF32 | ANZ52214.1_32 | APU00757.1_32 | 75.862 | Tail protein/Phage_TAC_7 |
| ORF33 | ANZ52215.1_33 | APU00758.1_33 | 81.818 | Tail protein/ Phage_P2_GpE superfamily |
| ORF34 | ANZ52216.1_34 | APU00759.1_34 | 51.981 | Tail length tape-measure protein/ tape_meas_TP901 |
| ORF35 | ANZ52217.1_35 | APU00760.1_35 | 81.379 | Tail protein |
| ORF36 | ANZ52218.1_36 | APU00761.1_36 | 77.465 | Tail protein/Phage_tail_X |
| ORF37 | ANZ52219.1_37 | APU00762.1_37 | 70.871 | late control protein GpD/  Phage_GPD superfamily |

| **ORF number** | **Ahp2 ORFs Accession** | **Phage 59.1 ORFs Accession** | **%identity** | **Annotation** |
| --- | --- | --- | --- | --- |
| ORF23 | ANZ52205.1_23 | APU00832.1_24 | 45.982 | Baseplate assembly protein V/  Phage_base_V superfamily |
|  |  | APU00833.1_25 |  |  |
| ORF24 | ANZ52206.1_24 | APU00834.1_26 | 64.035 | Baseplate assembly protein W/  GPW_gp25 superfamily |
| ORF25 | ANZ52207.1_25 | APU00835.1_27 | 65.95 | Baseplate assembly protein J/  Baseplate_J superfamily |
| ORF26 | ANZ52208.1_26 | APU00836.1_28 | 69.271 | Tail protein/Tail_P2_I |
| ORF27 | ANZ52209.1_27 | APU00837.1_29 | 49.419 | Tail fiber protein/  DUF3751 superfamily |
| ORF28 | ANZ52210.1_28 | APU00838.1_30 | 50.746 | Tail fibers |
| ORF29 | ANZ52211.1_29 | APU00839.1_31 | 42.553 | Hypothetical protein |
| ORF30 | ANZ52212.1_30 | APU00840.1_32 | 80.348 | Tail sheath/  FI domain |
| ORF31 | ANZ52213.1_31 | APU00841.1_33 | 75.148 | Major tail tube protein |
| ORF32 | ANZ52214.1_32 | APU00842.1_34 | 66.292 | Tail protein/Phage_TAC_7 |
| ORF33 | ANZ52215.1_33 |  |  | Tail protein/ Phage_P2_GpE superfamily |
| ORF34 | ANZ52216.1_34 | APU00843.1_35 | 51.14 | Tail length tape-measure protein/ tape_meas_TP901 |
| ORF35 | ANZ52217.1_35 | APU00844.1_36 | 64.706 | Tail protein |
| ORF36 | ANZ52218.1_36 | APU00845.1_37 | 63.38 | Tail protein/Phage_tail_X |
| ORF37 | ANZ52219.1_37 | APU00846.1_38 | 53.731 | late control protein GpD/  Phage_GPD superfamily |
