# Supplementary figures and images for: Molecular Characterization of Ahp2, a Lytic Bacteriophage of Aeromonas hydrophila
Source: Viruses. 2021 Mar 14;13(3):477. doi: 10.3390/v13030477 (PMC8001559; doi:10.3390/v13030477)

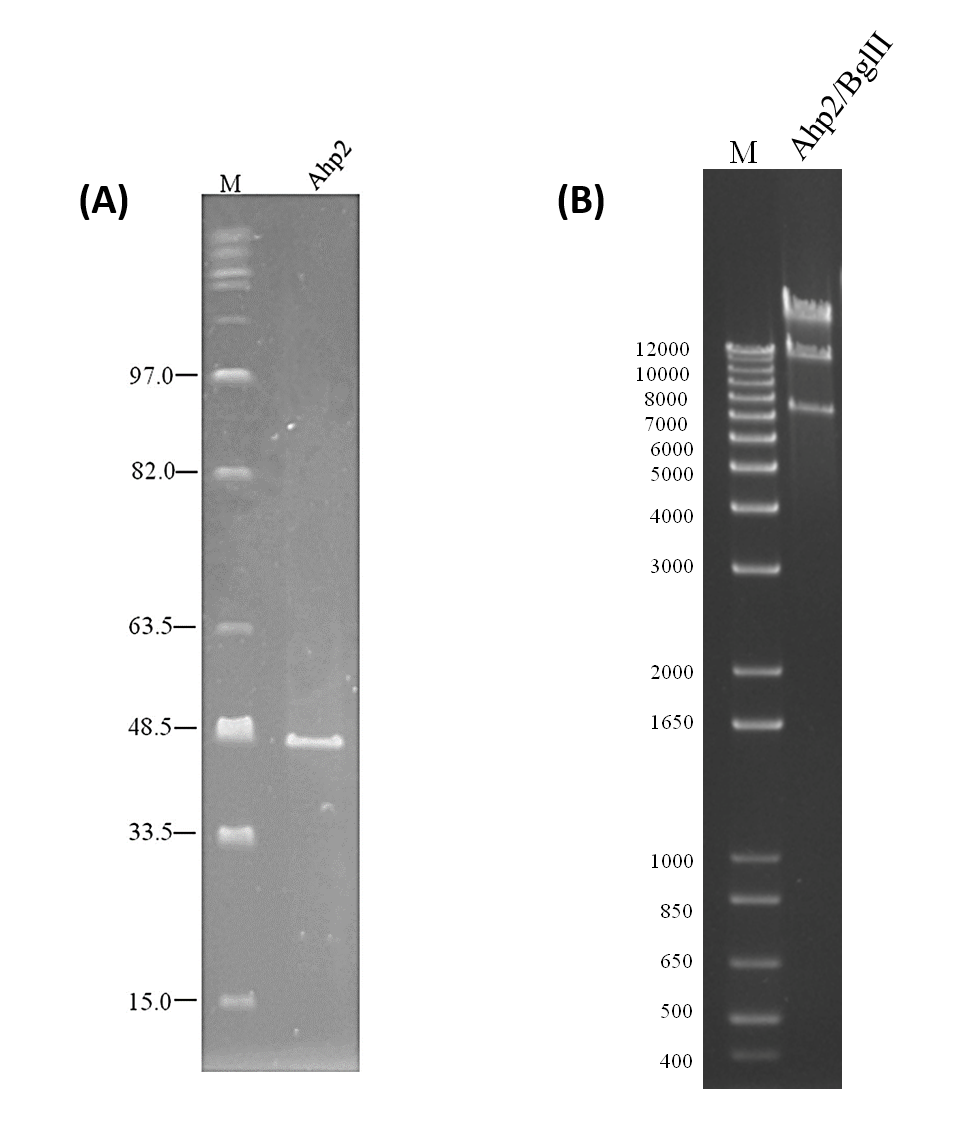

Supplement: Supplementary file 1 [file viruses-13-00477-s001.zip › Figure S1.png]
